# The upward shift of hilar structures and tracheal deviation in pleuroparenchymal fibroelastosis

**DOI:** 10.1186/s40248-019-0173-8

**Published:** 2019-03-07

**Authors:** Hiroshi Ishii, Yoshiaki Kinoshita, Hisako Kushima, Takashi Ogura, Kentaro Watanabe

**Affiliations:** 1 Department of Respiratory Medicine, Fukuoka University Hospital, 7-45-1 Nanakuma, Fukuoka, 814-0180 Japan; 2 Department of Respiratory Medicine, Kanagawa Cardiovascular and Respiratory Center, Yokohama, Kanagawa Japan

**Keywords:** Pleuroparenchymal fibroelastosis, Chest images, Upward shift of hilar structures, Tracheal deviation

## Abstract

The upward shift of hilar structures is a characteristic finding on chest radiographs in pleuroparenchymal fibroelastosis (PPFE). However, the relationship between the hilar shift and clinical parameters is unclear. In this study, the ratio of the length of the lung apex to the hilum and the length of the apex to the base of the right lung was measured using chest computed tomography (CT) at the time of the diagnosis, and the relationship with clinical parameters was investigated. We also examined the deviations of the trachea on chest radiographs and compared them with those in idiopathic pulmonary fibrosis (IPF) and early-stage lung cancer. Thirty-eight PPFE patients in a previous study included 20 patients who simultaneously showed the lower lobe lesions. The median ratio of the length of the apex to the hilum/apex to the base was 0.32 (range: 0.10–0.41) in PPFE, and this value was significantly lower than that in IPF (0.39; 0.32–0.45, *n* = 38) and in lung cancer (0.41; 0.33 to 0.45, *n* = 38) (*p* < 0.001, respectively). However, the ratio of the length of the apex to the hilum/apex to the base did not correlate with the dyspnea scale, body mass index or pulmonary function in PPFE. Tracheal deviations were observed in 41 out of 52 PPFE patients (36 with rightward deviations, 5 with leftward deviations) and in 30 out of 52 IPF patients (30 with rightward deviations) (*p* = 0.01). Although the existence of the upward shift of hilar structures on chest images might lead to a diagnosis of PPFE, the extent of hilar elevation does not necessarily reflect disease progression. Tracheal deviation is not a specific finding for PPFE.

## Background

The pleuroparenchymal fibroelastosis (PPFE) are located predominantly in the upper lobes [1, 2] with or without lower lobe lesions. The upward shift of the hilum [3, 4] are characteristic chest images findings of PPFE; however, the relationship between the hilar shift and clinical parameters is unclear.

We previously reported the characteristics of 52 patients with PPFE [5]. We showed that a low body mass index (BMI), decreased forced expiratory capacity (FVC) and increased ratio of residual volume (RV)/total lung capacity (TLC) in PPFE may be related to the progression of a flattened chest cage, which impairs distension of the chest cage at inspiration [5, 6].

In this study, we measured the extent of hilar elevation using chest images obtained at the time of the diagnosis to evaluate their relationship with other clinical parameters. In addition, we examined the deviations of the trachea and compared these findings with those in idiopathic pulmonary fibrosis (IPF) and/or in early-stage lung cancer as controls.

## Methods

**Fig. 1:**
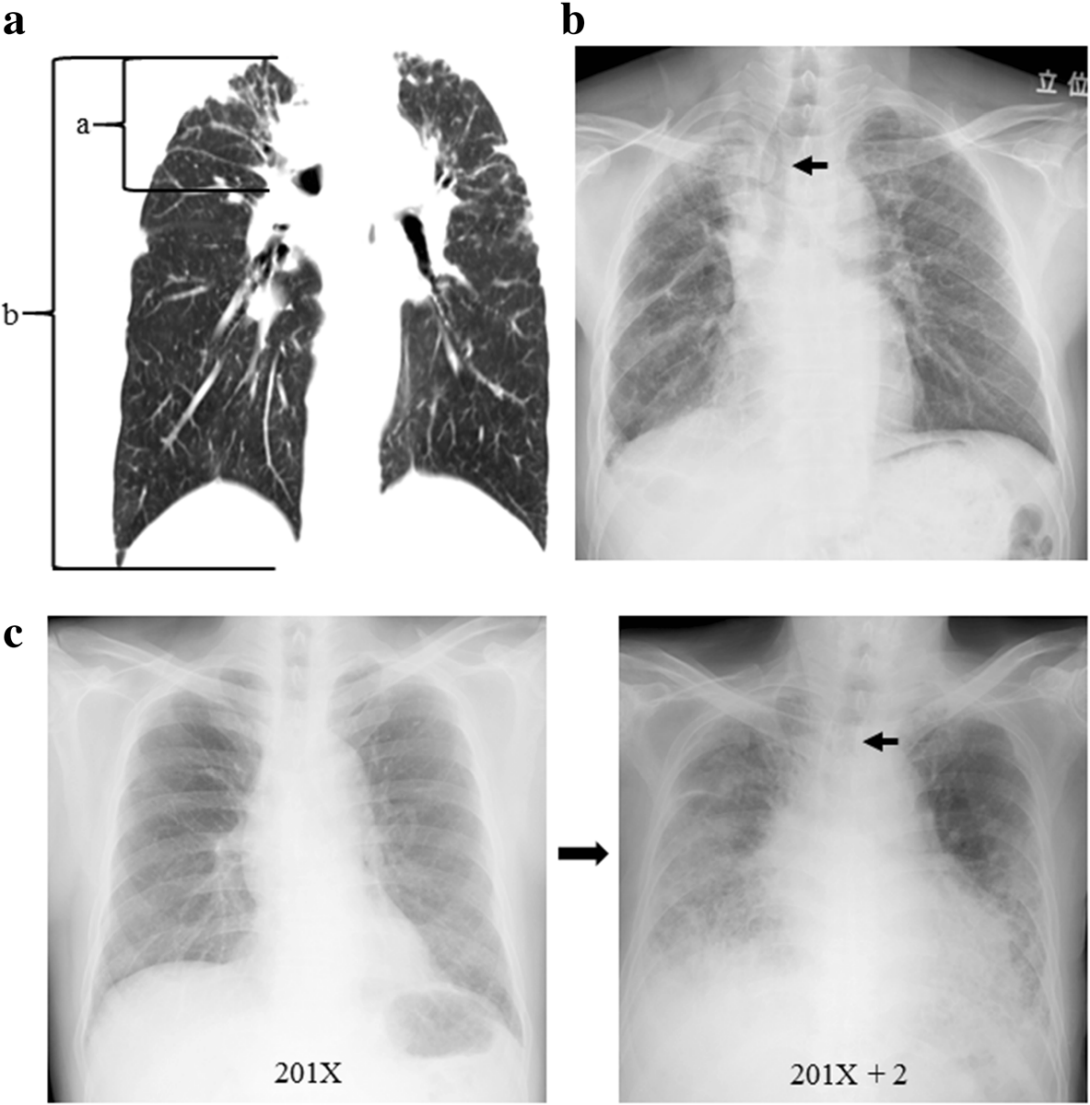
Coronal section image of chest computed tomography (CT) of a patient with PPFE (**a**). The ratio of the length of the lung apex to the hilum (a) and the length of the apex to the base (b) of the right lung was measured using axial section images on chest CT. Representative chest radiographs of rightward tracheal deviation (arrows) in a PPFE patient (**b**) and an IPF patient with the evolution of disease (**c**)

Of the 52 cases of PPFE diagnosed by multidisciplinary discussions [5], the whole lung field with high resolution computed tomography (CT) could be observed at the time of the diagnosis in 38 (22 males and 16 females, median age 62 years old). The ratio of the length of the lung apex to the hilum and the length of the apex to the base of the right lung was measured using axial images on chest CT. The position of the right hilum was defined as the point where the right main pulmonary artery flows into the right thorax (Fig. 1a). The clinical parameters used were as follows: the pulmonary functions, BMI (kg/m^2^), modified medical research council (MRC) breathlessness scale and gender, age and physiology score (GAP) score [7]. We examined the extent of hilar elevation using the same method in control groups: 38 cases of IPF (34 males and 4 females, median age 69 years old) and 38 cases of early-stage lung cancer without interstitial pneumonia who underwent HRCT for a staging evaluation (28 males and 10 females, median age 67 years old). All IPF patients were retrospectively confirmed to have met the criteria of the 2018 statement for IPF [8].

The tracheal deviations were examined in the 52 cases of PPFE and compared with those in 52 cases of IPF. In chest radiograph, cases with the left edge of the trachea located on the right side of midline were defined as rightward deviation, those with the right edge of the trachea located on the left side of midline were defined as the leftward deviation, and those with neither condition were regarded as having no deviation. Although the chest radiographs at the time of the diagnosis were used for the PPFE cases, the chest radiographs used for the IPF cases had been taken at any time.

## Results

The median ratio of the length of the apex to the hilum/apex to the base was 0.32 (range; 0.10 to 0.41, *n* = 38) in PPFE, which was significantly lower than that in IPF (0.39; 0.32 to 0.45, *n* = 38) and early-stage lung cancer (0.41; 0.33 to 0.45, *n* = 38) (*p* < 0.001 for both; unpaired *t*-test). The PPFE cases included 20 patients who simultaneously had other patterns of interstitial pneumonia in the lower lung lobes. The ratio of the length of the apex to the hilum/apex to the base did not correlate with any clinical parameters using Pearson’s correlation coefficient, including mMRC scale (*r* = 0.228, *p* = 0.169), BMI (*r* = 0.2136, *p* = 0.414), GAP score (*r* = 0.109, *p* = 0.526), RV/TLC (%pred.) (*r* = − 0.143, *p* = 0.459), FVC (%pred.) (*r* = 0.122, *p* = 0.48) and diffusing capacity of the lung for carbon monoxide (%pred.) (*r* = 0.196, *p* = 0.299).

The tracheal deviations (Fig.1b, c) were observed in 41 (79%) out of 52 PPFE patients (36 with rightward deviation, 5 with leftward deviation, 11 without deviation) and in 30 (58%) out of 52 IPF patients (30 with rightward deviation and 22 without deviation) (*p* = 0.01; χ^2^ test).

## Discussion

Upward shift of hilar structures is not rarely seen in daily practice. At that time, even if the case demonstrated an upper lobe-limited type of PPFE, followup might be terminated based on the judgment that such a finding is merely old pulmonary tuberculosis. Recently, PPFE cases with lesions other than in the upper lobe have steadily been reported, and not only idiopathic cases but also cases with some autoantibodies, cases in which hypersensitivity pneumonitis is suggested and cases associated with bone marrow or lung transplantation have been reported [9–12]. Upward shift of hilar structures associated with progressive contraction of the upper lobes is a well-known feature in PPFE, although cases with lesions other than in the upper lobe do not necessarily present with such findings.

All cases of PPFE examined in the present study had been histopathologically confirmed by a surgical lung biopsy, autopsy or lung transplantation. In other words, most cases required a histopathological diagnosis to elucidate the disease condition and to explore therapeutic options, and many cases had some lesions in the lower lung lobes. Our finding that a hilar elevation is not necessarily related to the clinical parameters suggests that the disease progression can alter the position of the hilum, depending on the time of the diagnosis, especially in PPFE cases with lower-lobe lesions. However, the present study is associated with several limitations, including its retrospective nature, small number of patients, and wide variation in the time of the diagnosis in both PPFE and IPF cases. In the present study, the right hilum was selected for the evaluation, as the point at which the right main pulmonary artery flows into the right thorax is easy to detect on HRCT and well reflects the position of the right hilum on chest radiographs. The use of chest radiograph would be enough to detect the presence of hilar elevation in clinical practice.

To our knowledge, this is the first study to focus on the tracheal deviation in PPFE. The trachea is frequently flexed and deviated to the right in association with contraction of upper lobes due to disease progression. However, we found that the rightward deviation of the trachea was frequently observed in advanced IPF cases, indicating that the rightward deviation is not necessarily a finding specific to PPFE. As a potential cause of the large number of cases of right deviation, both PPFE and IPF can show lesions predominantly in a single lung; it was reported that asymmetrical IPF may be related to locoregional factors, such as gastroesophageal reflux [13]. In addition, when the contraction of the lung occurs, the trachea can be predisposed to anatomically and structurally be flexed and deviated to the right.

## Conclusion

Although the presence of hilar elevation may be a trigger for detecting not only old pulmonary tuberculosis but also PPFE, chest physicians should be aware that the hilar elevation in patients with the lower lobe lesions may not be noticeable or inconspicuous due to shrinkage of the whole lung with disease progression. Further prospective comparative studies targeting a larger cohort, including patients with PPFE and others, will be required to corroborate and expand upon our findings.

## Abbreviations

%pred: Percentages of predicted values
BMI: Body mass index
CT: Computed tomography
FVC: Forced vital capacity
GAP: Gender, age and physiological variables
IPF: Idiopathic pulmonary fibrosis
mMRC: Modified medical research council
PPFE: Pleuroparenchymal fibroelastosis
RV: Residual volume
TLC: Total lung capacity
